# Hyaluronan supports the limbal stem cell phenotype during ex vivo culture

**DOI:** 10.1186/s13287-022-03084-8

**Published:** 2022-07-30

**Authors:** Sudan Puri, Isabel Y. Moreno, Mingxia Sun, Sudhir Verma, Xiao Lin, Tarsis F. Gesteira, Vivien J. Coulson-Thomas

**Affiliations:** 1 College of Optometry, University of Houston, Houston, TX USA; 2 Department of Zoology, Deen Dayal Upadhyaya College, University of Delhi, New Delhi, India

**Keywords:** Limbal epithelial stem cells, Hyaluronan, Extracellular matrix, Limbal stem cell culture, Glycosaminoglycans

## Abstract

**Background:**

Hyaluronan (HA) has previously been identified as an integral component of the limbal stem cell niche in vivo. In this study, we investigated whether a similar HA matrix is also expressed in vitro providing a niche supporting limbal epithelial stem cells (LESCs) during ex vivo expansion. We also investigated whether providing exogenous HA in vitro is beneficial to LESCs during ex vivo expansion.

**Method:**

Human LESCs (hLESCs) were isolated from donor corneas and a mouse corneal epithelial progenitor cell line (TKE2) was obtained. The HA matrix was identified surrounding LESCs in vitro using immunocytochemistry, flow cytometry and red blood exclusion assay. Thereafter, LESCs were maintained on HA coated dishes or in the presence of HA supplemented in the media, and viability, proliferation, cell size, colony formation capabilities and expression of putative stem cell markers were compared with cells maintained on commonly used coated dishes.

**Results:**

hLESCs and TKE2 cells express an HA-rich matrix in vitro, and this matrix is essential for maintaining LESCs. Further supplying exogenous HA, as a substrate and supplemented to the media, increases LESC proliferation, colony formation capabilities and the expression levels of putative limbal stem cell markers.

**Conclusion:**

Our data show that both exogenous and endogenous HA help to maintain the LESC phenotype. Exogenous HA provides improved culture conditions for LESC during ex vivo expansion. Thus, HA forms a favorable microenvironment for LESCs during ex vivo expansion and, therefore, could be considered as an easy and cost-effective substrate and/or supplement for culturing LESCs in the clinic.

**Supplementary Information:**

The online version contains supplementary material available at 10.1186/s13287-022-03084-8.

## Introduction

The cornea is the anterior, transparent, and avascular part of the eye with high refractive power that is essential for normal vision. The corneal epithelium is the outermost layer of the cornea and, as such, is exposed to the external environment, being prone to physical and chemical injuries. Defects in the ability to repair and restore epithelial integrity after injury can lead to loss of corneal transparency and visual impairment. The self-renewing properties of the corneal epithelium are important requirements for corneal integrity and function. In the human cornea, LESCs exist within the basal epithelial layer of crypts, the palisades of Vogt, in the limbal region [1–6]. These crypts are not present in the mouse cornea [7]; however, numerous studies have shown that LESCs are located in the basal layer within the limbal region [8–12]. LESCs undergo asymmetric cell division producing a stem cell that remains in the limbal region and a transient amplifying cell (TAC) that migrates centripetally toward the central cornea while differentiating into a corneal epithelial cell [3, 13]. Damage or loss of LESCs can lead limbal stem cell deficiency (LSCD), characterized by a reduced ability to repopulate the corneal epithelium after injury [14–16]. LSCD is an important medical condition with clinical manifestations ranging from corneal opacification, vascularization and severe pain, to complete loss of vision in more severe cases [17–19].

Recently, treatment options for LSCD have included cultivated limbal epithelial transplantation (CLET), which involves isolating LESCs from the patient’s contralateral eye or from human leukocyte antigen (HLA) matched donor corneas and expanding these cells ex vivo before transplanting them onto the LESC deficient cornea [20–22]. To date, several studies have investigated methods of expanding LESCs ex vivo while maintaining their stem cell phenotype; however, an optimal substrate for supporting these cells ex vivo has still not been identified [7, 21, 23–28]. Due, in part, to the lack of proper guidelines in optimal culture conditions of LESCs for CLET, the success rate of LESCs transplantation has been largely variable [17, 19, 21, 22]. Amniotic membrane is commonly used as a scaffold for expanding LESCs ex vivo [29, 30], however, the main drawbacks for this method are the inter-membrane variability, cost and difficulty implementing this procedure throughout the world. The amniotic membrane is a hyaluronan (HA)-rich tissue, and an HA-complex (HA/HC-TSG-6) purified from the amniotic membrane has been shown to support LESCs ex vivo [31, 32]. This same complex has also been shown to have both anti-inflammatory and anti-scarring properties [33]. Thus, specifically the HA-complex within the amniotic membrane is believed to provide the therapeutic benefits during CLET. This same HA-complex is synthesized by human umbilical cord mesenchymal stem cells (hUMSCs) and embryonic MSCs, and also plays a role in supporting these cells ex vivo which is necessary for engraftment success [34, 35].

Research has demonstrated that a specific microenvironment exists in the limbal region surrounding the LESCs, specifically a LESC niche (LSCN) in vivo [24, 28, 36–38]. The extracellular matrix (ECM) composition of the LSCN is different to the ECM that is present in the peripheral cornea, central cornea and conjunctiva, and provides a unique microenvironment that is necessary for maintaining viable LESCs [39–43]. Our group recently showed that the LSCN is made up of a HA-rich matrix which is necessary to maintain viable LESCs, and knock-out mice lacking HA biosynthetic enzymes in the limbus also lack LESCs [38]. In addition, HA has also been shown to be present in the LSCN of human, pig, bovine and rabbit corneas [44–46]. Herein we investigated first whether LESCs continue to express HA while cultured in vitro, and, also, verify whether this endogenous HA matrix supports the LESCs during ex vivo expansion. Thereafter, we investigated whether exogenous HA could also have the capability to further support viable LESCs in vitro.

## Methods

### Human LESC isolation

Full thickness corneas from human donor eyes (healthy tissue however deemed unsuitable for transplantation) with a mean age of 42.9 ± 8.1 years were obtained from Saving Sight Inc., Kansas City, MO, USA and Miracles in Sight, Winston-Salem, NC, USA. The use of human tissues adhered to the tenets of the Declaration of Helsinki and were used with prior research consent. Upon arrival, the corneas were washed in Hanks’ balanced salt solution (HBSS) (Gibco, Life Technologies, Carlsbad, CA, #14170-112) containing antibiotic–antimycotic (Gibco, Life Technologies, Carlsbad, CA). With the aid of a dissecting microscope placed in a horizontal laminar flow hood, the limbal rim was dissected away from the remaining cornea. Each limbal rim was enzymatically digested with 1.53 g/ml Dispase II (1.56U/mg, #D4693-1G, Sigma, USA) and 2 mg/ml Collagenase A from *Clostridium histolyticum* (0.212 U/mg, #10103586001, Roche, Germany) at 37 °C for 15 min to generate loose epithelial sheets which were gently peeled off and further dissociated into single cells by digestion with 0.25% trypsin–EDTA (#25200-056, Gibco, Life Technologies, Carlsbad, CA) at 37 °C for 10 min. Single cell suspensions were seeded onto pre-coated culture plates (Corning, Tewksbury, MA) in the presence or not of 3T3 feeder cells. The cells were cultured in EpiLife culture medium supplemented with bovine pituitary extract, epidermal growth factor (Cascade Biologics, Portland, OR, USA) and 1X penicillin–streptomycin–amphotericin B (Gibco, Life Technologies, Carlsbad, CA, #15240–062) or DMEM/F12 (Dulbecco's Modified Eagle Medium/Nutrient Mixture F-12) SHEM media (Gibco, Life Technologies, Carlsbad, CA, #11330-032) supplemented with 50ug/mL of gentamycin (Gibco, Life Technologies, Carlsbad, CA, #15750-060), 5% fetal bovine serum (FBS) (Gibco, Life Technologies, Carlsbad, CA, USA), 1X insulin transferrin selenium (ITS) (Sigma, #I3146, usa), 05.ug/mL of hydrocortisone (Sigma, #H2270, USA), 5 ng/mL of epidermal growth factor (EGF) (Sigma, #E9644, USA), and 30 ng/mL of cholera toxin (Sigma, USA, C8180) and incubated at 37 °C under 5% CO_2_ and 95% humidity, media was changed every other day. Prior to seeding hLESCs, 3T3 cells were seeded into the pre-coated dishes at a density of 2 × 10^3^ cells per cm^2^ and allowed to adhere for 4 h after which they were treated with 5 µg/mL of Mitomycin C (Millipore, Billerica MA, USA) for 2 h. The cells were subcultured at a ratio of 1:3 using 0.25% Trypsin EDTA. Unless mentioned otherwise, all experiments were carried out with hLESCs at passage 3. Throughout the study, twenty separate LESC batches were prepared, using ~ 3 to 7 pooled corneas per preparation to ensure reproducibility of our findings. The 3T3 feeder cells were maintained in DMEM media supplemented with 10% FBS and 1X antibiotic–antimycotic (Gibco, Life Technologies, Carlsbad, CA, USA) at 37 °C and 5% CO_2_.

### Mouse TKE2 cell line

Mouse TKE2 limbal/corneal epithelium-derived progenitor cell line [47] was kindly provided by Dr. Chia-Yang Liu (Indiana University Bloomington, Bloomington, Indiana, USA). Cells were maintained in Defined Keratinocyte Serum-Free Media (dKSFM; Life Technologies, Carlsbad, CA, USA) supplemented with 100 ng/ml cholera toxin (Millipore, Billerica MA, USA), 10 ng/ml recombinant EGF, and 1X antibiotic–antimycotic solution (Gibco, Life Technologies, Carlsbad, CA, USA) and media changed every other day. Cells were subcultured at a ratio of 1:3 using TrypLE Express enzyme (Gibco Life Technologies, Carlsbad, CA, USA). In order to induce differentiation of TKE2 cells, the culture medium was changed from dKSFM to DMEM (Gibco, Life Technologies, Carlsbad, CA, USA) supplemented with 10% FBS and 1X antibiotic–antimycotic solution.

### Immunocytochemistry

For immunocytochemistry, cells were cultured under different conditions for 48 h and fixed in 2% paraformaldehyde for 30 min. Cells were treated with 0.1 M glycine for 1 min and then blocked and permeabilized with 10% FBS solution containing 0.01% saponin for at least 2 h. Thereafter, cells were incubated with primary antibodies or biotinylated HA binding protein (HABP, Millipore, USA). To evaluate the number of LESCs within the cultures, antibodies for putative stem cell markers K19 (ab166857; Abcam, Cambridge, MA, USA), K15 (ab185627; Abcam, Cambridge, MA, USA) and ΔNp63 (ab166857; Abcam, Cambridge, MA, USA) were used. In order to analyze CD44 expression, anti-CD44 (5D2-27, DSHB) was used. The cells were then washed and incubated with relevant secondary antibodies conjugated with Alexa®488 or Alexa®555, or with NeutrAvidin®555 in the case of biotinylated HABP. Thereafter, nuclei were labeled with DAPI, cytoskeleton labeled with Phalloidin®Alexa Fluor 647 (ThermoFisher Scientific, Eugene, OR) and coverslips mounted with Fluormount-G® (Southern Biotechnology Associates, USA). The immunolabeled cells were scanned under an LSM 800 confocal microscope using the Zen Image software (Zeiss) and quantified using ImageJ software (National Institutes of Health, Bethesda, MD).

### Flow cytometry

hLESCs were analyzed by flow cytometry in order to evaluate the co-localization rate between HA and LESC markers. For such, single cell suspensions (0.5 × 10^6^ cells) were incubated with anti-K15 antibody or anti-ΔNp63 antibody and biotinylated HA binding protein (Millipore—HABP) in 100 µl PBS containing 2% FBS for 40 min, washed with PBS, and then incubated with donkey anti-mouse conjugated with Alexa Fluor®488 and avidin conjugated with AlexaFluor®555 for 30 min. Cells were analyzed and quantified using FACSCanto II (BD, Biosciences, Bedford, MA, USA) flow cytometer and FlowJo V10 software (Tree Star, Ashland, OR, USA).

### Red blood cell exclusion assay

LESCs were isolated and cultured as outlined above and when cells reached approximately 60% confluence, fresh mouse red blood cell suspensions prepared as previously described [48], were placed over the cells and left to settle for 30 min at 37 °C. The cells were imaged under an EVOS cell imaging system (ThermoFisher Scientific, USA) and five representative images captured per well in a blinded manner. The exclusion space was measured by calculating the thickness of the space devoid of cells for five LESCs per field in fifteen fields per experimental condition, also in a blinded manner. In order to ascertain the exclusion space represents the pericellular HA matrix, LESCs were treated or not with a combination of Hyaluronidase from *Streptomyces hyalurolyticus* (Hyalase, Sigma-Aldrich, USA, #H1136), enzyme that cleaves HA, and 4-Methylumbelliferone (4MU, Sigma-Aldrich, USA, #M1381), an inhibitor of HA biosynthesis, prior to the placement of the red blood cells. The experiment was carried out in triplicate and repeated twice.

### Analysis of the role of the endogenous HA matrix present surrounding hLESCs and TKE2 cells

In order to elucidate the role of endogenous HA on LESCs, both TKE2 cells and hLESCs were treated with a combination of hyalase and 4MU. For such, LESCs were treated with a combination of 1 mM 4MU and 3U/ml hyalase, referred to as [Hyalase + 4MU]. In our laboratory, we found that a combination of 1 mM 4MU and 3U/ml hyalase reduces extracellular HA by more than 85% within 24 h of treatment (results not shown). In order to verify the mechanism by which HA supports LESCs, anti-CD44 neutralizing antibody (R&D systems, Minneapolis, USA#AF6127) was added to the culture media in order to block the binding of HA to CD44. Importantly, CD44 contains a HA binding extracellular domain and the anti-CD44 neutralizing antibody used in this study has been shown to block the binding of HA to its major cell surface receptor CD44 (R&D systems, Minneapolis, USA, AF6127).

### Coating culture dishes

In order to test the effect of HA as a substrate for culturing human and mouse LESCs ex vivo, culture dishes and coverslips were pre-coated with HA, and compared to dishes coated with other commonly used substrates, such as collagen IV (Col IV) and fibronectin (FNC). In order to increase the binding of HA to the polystyrene culture dish, positively charged poly-l-Lysine (PLL) was used to enhance electrostatic interaction of negatively charged HA to the culture plates, thus PLL/HA coated dishes, as previously shown [49]. For such, culture dishes or coverslips were previously coated with PLL solution (Sigma, USA, #P9156) for 4 h at room temperature or overnight at 4 °C and, thereafter, washed 4 times with PBS. Subsequently, the previously PLL coated plates and coverslips were washed and coated with 0.1% HA (Lifecore biomedical, Minnesota, USA) for 4 h at room temperature or overnight at 4 °C and, thereafter, washed four times with PBS. These coated dishes are hereafter referred to as PLL/HA coated dishes. Given that PLL supports the attachment of cells to culture dishes, we also included PLL alone as a control substrate in the study. For such, culture plates or coverslips were coated with PLL solution for 4 h at room temperature or overnight at 4 °C and, thereafter, washed 4 times with PBS, yielding PLL coated dishes. For FNC coated dishes and coverslips, they were covered with 200 µl/cm^2^ FNC Coating Mix® (AthenaES, USA, #0407) for at least 30 s and culture dishes washed four times with PBS, according to manufacturer’s recommendations. FNC Coating Mix® is a commercially available culture reagent containing bovine fibronectin 10 µg/ml, Type I Collagen 35 µg/mL, and serum albumin 1000 µg/ml, used to enhance the attachment of adherent cells to culture dishes. Thereafter, for FNC/HA coatings, dishes were subsequently coated with 0.1% HA for 4 h at room temperature or overnight at 4 °C and, subsequently, washed four times with PBS. For coating culture dishes or coverslips with Col IV, they were treated with Col IV coating solution (20 µg/ml) prepared in 0.2% acetic acid, for 4 h at room temperature or overnight at 4 °C and, thereafter, washed four times with PBS, yielding Col IV coated dishes. For Col IV/HA coatings, dishes were subsequently coated with 0.1% HA for 4 h at room temperature or overnight at 4 °C and subsequently washed four times with PBS.

### Analysis of the effect of exogenous HA on TKE2 cells and hLESCs

In order to elucidate the role of exogenous HA on LESCs, firstly both TKE2 cells and hLESC were treated with HA of different molecular weights. For such, HA treatments consisted of 0.01% HMWHA (Sodium Hyaluronate, Lifecore Biomedical, 1.55MDa MW, #HA15M-5), 0.01% LMWHA (R&D Systems, Minneapolis, USA, #GLR001,) or 0.01% ULMWHA (R&D Systems, Minneapolis, USA, #GLR003), added to the media in order to evaluate the effect of exogenous soluble HA on LESCs, referred to as [HMWHA], [LMWHA], or [ULMWHA], respectively. The molecular weight of the HA standards used in this study (HMWHA, LMWHA and ULMWHA) was verified in our laboratory and were consistent with the values stated by the manufacturer (results not shown).

### Cell viability assay

3-(4,5-dimethylthiazol-2-yl)-2,5-diphenyltetrazolium bromide (MTT) assay was used to determine the effect of HA on the viability of LESCs in vitro. For such, cells were seeded at 2 × 10^4^ cells per well in 96-well plates pre-coated as outlined above or on uncoated dishes in the case of TKE2 cells controls. It is important to note that hLESCs do not adhere well to uncoated dishes and, therefore, uncoated dishes were not used as controls for hLESCs. In order to study the effects of exogenous and/or endogenous HA on LESCs, HA of different sizes, hyalase and 4MU or anti-CD44 were added to the media 4 h after seeding the cells, as outlined above. 48 h after seeding the cells, 5 mg/ml of MTT stock solution (Sigma-Aldrich, #M5655, USA) was added to each well to a final concentration of 0.5 mg/ml, and the plate was incubated for an additional 4 h. Subsequently, the MTT solution was replaced with dimethyl sulfoxide (DMSO—solubilizing solution, Sigma-Aldrich, USA, #D8418) and incubated at room temperature or 37 °C for 30 min to 2 h (until cells were lysed and purple formazan crystals were dissolved). Absorbance was quantified by measuring optical density (OD) at 540 nm using a microplate reader (FLUOstar Omega; BMG Labtech, Ortenberg, Germany). This experiment was carried out with five replicates per experimental point and repeated at least three times for each cell type.

### Cell adhesion assay

TKE2 cells and hLESCs were seeded at 2 × 10^4^ cells per well in 96-well plates pre-coated or not, as outlined above. Cells were left to adhere for 60 min at 37 °C and 5% CO_2_ and thereafter washed with PBS to remove any non-adherent cells. Images were captured using an EVOS microscope and number of cells attached to the different substrates quantified using ImageJ software (NIH, Bethesda, MD). Data is represented as the number of cells per unit area calculated from the average of five replicates. This experiment was carried out three times in triplicate.

### Cell proliferation assay

Cell proliferation was measured using a BrdU (5-bromo-2′-deoxyuridine) Cell Proliferation Assay Kit (EMD Millipore, MA, #2750), according to the manufacturers’ instructions. For such, hLESCs or TKE2 cells were seeded at a density of 5000 cells per well in 96-well plates and left for 48 h at 37 °C and 5% CO_2_. BrdU was added to the culture media and plates incubated for a further 8 h. Following this incubation period, the cells were fixed using the provided fixing solution. To detect the BrdU-incorporated cells, anti-BrdU monoclonal antibody was added, followed by the addition of IgG-peroxidase conjugated secondary antibody, substrate and stop solution according to the manufacturers’ instructions. Absorbance was measured as OD at 450 nm using a microplate reader (FLUOstar Omega; BMG Labtech, Ortenberg, Germany). This experiment was carried out with five replicates per experimental point and repeated at least three times for each cell type.

### Colony formation assay

An important aspect of stem cells is their ability to form holoclones in vitro, as previously shown [47, 50]. Thus, a colony formation assay was used to evaluate the “stemness” of stem cells under different conditions. For such, hLESCs or TKE2 cells were seeded onto pre-coated 6-well plates at a low density of 1 × 10^3^ cells per cm^2^ in the presence or not of 3T3 feeder cells. The plates were incubated at 37 °C and 5% CO_2_ for 13 days to allow for the formation of colonies. After 13 days, the media was removed, and plates rinsed with PBS. Colonies were stained with 0.5% crystal violet (w/v) prepared in buffered paraformaldehyde 2% (v/v) for at least 30 min. After carefully removing the crystal violet solution, the plates were rinsed with water and left to air dry at room temperature. The number of stained “holoclone-like” colonies (compact, homogenous and rounded colonies with smooth perimeters) larger than 1000 μm diameter was counted and data presented as the total number of “holoclone-like” colonies formed. Paraclones and merocolones were identified and were not included in the colonies that were counted. The experiments were repeated independently at least three times for TKE2 cells and hLESCs.

### In vitro scratch assay

TKE2 cells were seeded at 2 × 10^4^ cells per well in 24-well plates and cultured until they formed a confluent monolayer. Thereafter, a single scratch was made down the center of the well by scraping the cells with a 200 µL sterile pipette tip and the wells were subsequently washed with sterile PBS to remove any loose cells and debris. Thereafter, the cells were cultured with 0.01% HA or 1 mM 4MU + 3U/mL Hyalase. PBS served as the vehicle control. Images were taken at different time-points, specifically 0 h, 6 h, 18 h, 24 h and 42 h after the scratch and the cell-free area quantified using ImageJ. The rate of wound healing was calculated as a percentage of scratched area over time (Scratched area at a certain time point/Scratched area at 0 h) × 100. The experiment was carried out in quadruplicate and repeated independently at least three times.

### mRNA extraction and quantitative real-time PCR (qPCR) analysis

Total mRNA was isolated from the cells using Trizol Reagent (Invitrogen, Carlsbad, CA, USA) according to the manufacturer’s recommendations. Nanodrop spectrophotometry (NanoDrop ND-1000 Technologies Inc., Wilmington, DE, USA) 260/280 ratio was used to determine RNA concentration and purity. Complementary DNA (cDNA) was synthesized from 2.0 μg of total RNA using the high-capacity cDNA Reverse Transcription kit (Applied Biosystems, Lithuania, #4368814) according to the manufacturer’s instructions. Quantitative real-time PCR amplification was carried out using 50 ng of the cDNA (1:5) and the Powerup SYBR Green Master Mix kit (Applied Biosystems, Lithuania, #A25918) using a C1000TM Thermal Cycler, CFX96TM Real-Time System (Bio-Rad, California, USA). PCR reactions were run in triplicates using primer sequences designed for specific genes. The primers used for hLESCs were KRT12 forward CTGTGGAGGCCTCTTTTCTG and reverse ATTCCAGCTATCCCCAATCC, KRT3 forward TTGACTGCCGCAGAGTTCAT, reverse ATGAAGACCTTGGATCTGCACG, KRT15 forward CCACGAAGAGGAGATGAAGGAG, reverse CACGGGTCAGGTCCACAC, ΔNp63 forward GTAACACTAGGGCCTTGGAAA, reverse CATGAGGAGGTAAGCCAGTAAG, KRT19 forward ATATGAGGTCATGGCCGAGC and reverse GGTTCAATTCTTCAGTCCGGC, GAPDH forward CTGCCGGTGACTAACCCTG and reverse GCCCAATACGACCAAATCAGAGA, *β*-actin forward CCCTATAAAACCCAGCGGCG and reverse TCATCATCCATGGTGAGCTGG. The PCR protocol consisted of an activation cycle of 95 °C for 10 min followed by 40 cycles of 95 °C for 15 s and 60 °C for 1 min. GAPDH and *β*-tubulin/*β*-actin were used as housekeeping genes to normalize the gene expression levels which were calculated by the comparative CT method (− 2^ΔΔCT^). Genes were considered as differentially expressed when their expression levels exceeded a twofold difference. The experiment was carried out in triplicate and repeated independently at least three times.

### Cell senescence

In order to investigate whether culturing LESCs with HA could prevent or delay senescence in cultured LESCs, hLESCs were cultured onto differently coated coverslips at a seeding density of (2.4 × 10^4^ cells/mL) with and without the addition of 0.01% HA into the culture media. Cells were maintained in EpiLife media supplemented with 10% human corneal growth serum (HCGS) (Gibco, Life Technologies, Carlsbad, CA, USA) and 1X antibiotic–antimycotic. Cells were maintained until passage 11 and the Senescence *β*-Galactosidase Staining Kit (Cell Signaling Technology, MA, USA, #9860) was used at the end of each passage from passage 6, in order to identify when cell senescence is present, following the standard protocol. In short, culture media was removed from the cells, cells were rinsed with 1X PBS, and fixed with 2% paraformaldehyde. Fixed cells were stained with the *β*-galactosidase staining solution and incubated at 37 °C overnight. Coverslips were mounted and imaged under a light microscope (Leica) at 10× magnification and senescence determined by the identification of a blue color within cells.

### Statistical analysis

Statistical analyses were performed using the statistical package within GraphPad Prism version 6.0 (GraphPad Software, San Diego, CA, USA) and data expressed as mean ± standard deviation (SD) of the mean from individual experiments. The image quantification and analysis were masked to avoid bias whenever possible. Differences between two experimental points were evaluated using the *t* test and for multiple comparisons by analysis of variance (ANOVA). *p* ≤ 0.05 was considered as statistically significant.

## Results

### LESCs express an HA-rich matrix in vitro

**Fig. 1:**
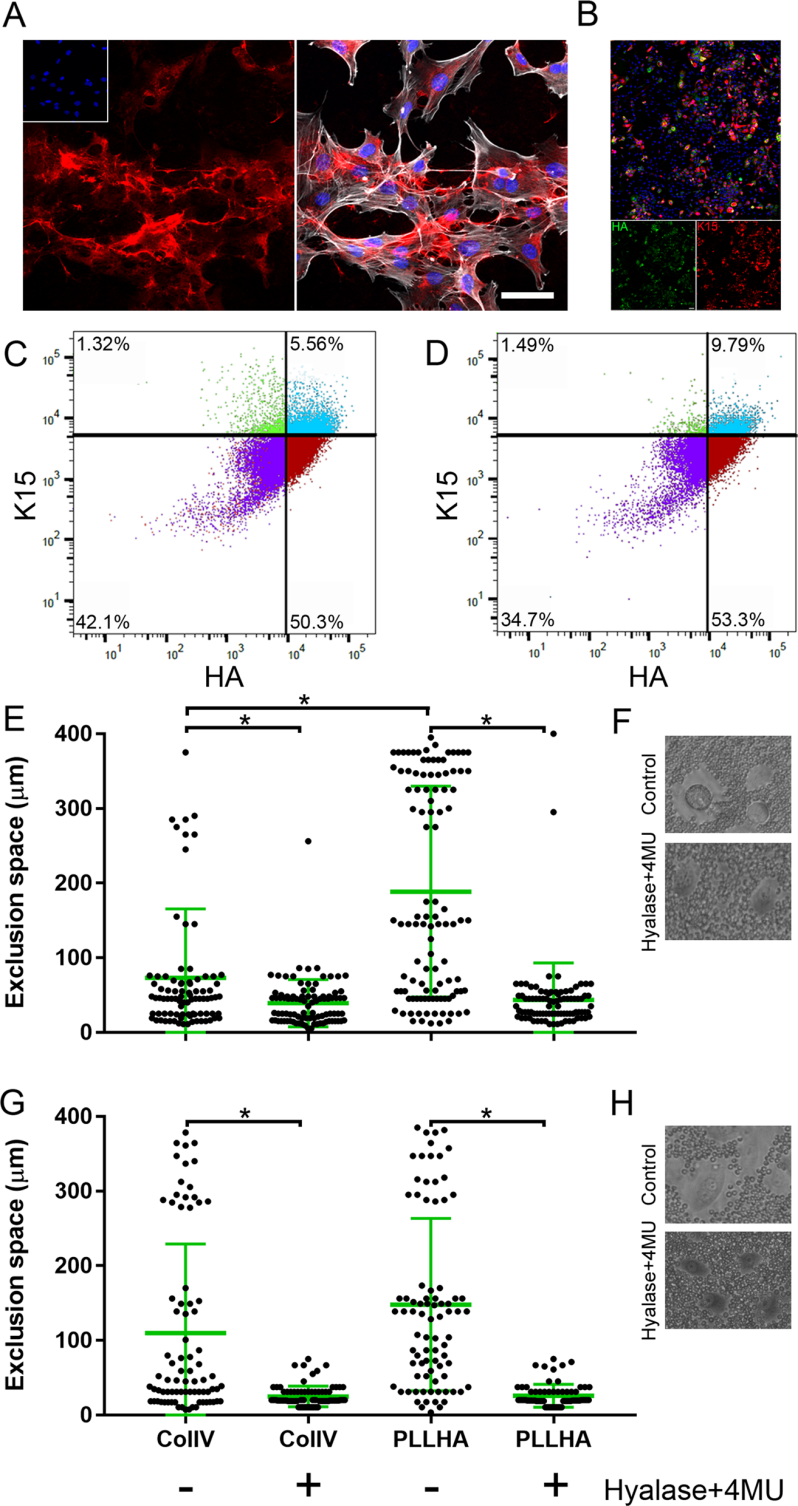
LESCs express HA in vitro. **A** hLESCs were cultured in vitro and the expression and localization of HA (red) and Phalloidin (white) investigated. Scale bar represents 50 μm (**B**) hLESCs were cultured in vitro and the expression and localization of HA (green) and K15 (red) investigated showing there is a strong correlation between the expression of putative limbal stem cell marker K15 and HA. Specifically, 97% of cells expressing K15 also express HA. **C** and **D** The expression of HA was also analyzed by flow cytometry after cells were maintained on PLL (**C**) or PLL/HA (**D**) coated dishes. There is a linear relationship between the expression of HA and K15, therefore cells that express high levels of K15 also express HA and cells that do not express K15 also do not express HA. **E**, **H** The expression of an HA matrix by hLESCs was analyzed using the red blood exclusion assay and the exclusion space measured. This assay evidences that a subset of both circular and non-circular cells express a thick glycocalyx and that the removal of HA from the ECM precludes the formation of this glycocalyx, indicating HA is necessary for the formation of a hLESC specific ECM (glycocalyx). *Represents *p* ≤ 0.05, scale bar represents 1000 μm

Our group has previously shown that the murine LSCN presents an HA-rich matrix, and this matrix is essential for maintaining LESCs in the “stem cell” state [38]. Our unpublished findings show a similar HA matrix is also present in the human LSCN [46]. Therefore, we investigated whether LESCs also express an HA matrix when cultured in vitro. We found that hLESCs express an organized HA matrix in vitro (Fig. 1A, B). Interestingly, we found a high co-localization rate between cells that expressed putative stem cells markers and the HA matrix, thus, it is specifically LESCs that present a pericellular HA matrix in culture (Fig. 1A). A 97% co-localization rate was observed for cells expressing both K15 and HA, and a 91% co-localization rate was observed for cells expressing both ΔNp63 and HA (Fig. 1B and images not shown, respectively). We also investigated whether TKE2 cells express an HA-glycocalyx and found that indeed mouse limbal progenitor epithelial cells also express an HA-rich matrix in culture. Thereafter, the co-localization of HA and putative stem cells markers was also analyzed by flow cytometry. Interestingly, there is a linear relationship between the expression of K15 and HA by flow cytometry (Fig. 1C, D). Around 5.56% of hLESCs seeded on PLL and ~ 9.79% of hLESCs seeded on PLL/HA expressed both high levels of K15 and HA, respectively (upper right quadrant, Fig. 1C, D). Thus, seeding hLESCs on PLL/HA increases the number of K15^HIGH^/HA^HIGH^ cells by approximately two fold (Fig. 1C, D). This data indicates that the HA glycocalyx could be used as a LESC marker in vitro, which could be useful as an extracellular marker for cell sorting. The pericellular HA matrix is closely associated with the hLESC membrane, and, therefore, could also be referred to as a HA-rich glycocalyx. In order to further characterize the HA matrix, we carried out the red blood cell exclusion assay. The red blood cell exclusion assay involves allowing red blood cells to settle at the bottom of a culture dish containing previously plated cells. The presence of a rich glycocalyx surrounding cells prevents the red blood cells from approaching the cell membrane, and this can be quantified as the exclusion space representing the area containing a pericellular matrix. The exclusion space surrounding LESCs was compared to that around hLESCs that had been previously treated with Hyalase and 4MU, in order to specifically quantify the HA glycocalyx (Fig. 1E–H). Hyalase + 4MU treatment, which specifically reduces pericellular HA, eliminated the exclusion space, indicating removing HA from around LESCs abrogates the assembly of their glycocalyx (Fig. 1E–H). The exclusion space was quantified separately around rounded and non-rounded cells in LESC cultures that were maintained on either ColIV or PLL/HA coated dishes (Fig. 1E–H). Our data show that a subset of rounded and non-rounded cells within our LESCs cultures presented a HA glycocalyx, and this population is greater in cells maintained on PLL/HA coated dishes when compared to ColIV coated dishes (Fig. 1E–H).

### Effect of HA on viability of LESCs

**Fig. 2:**
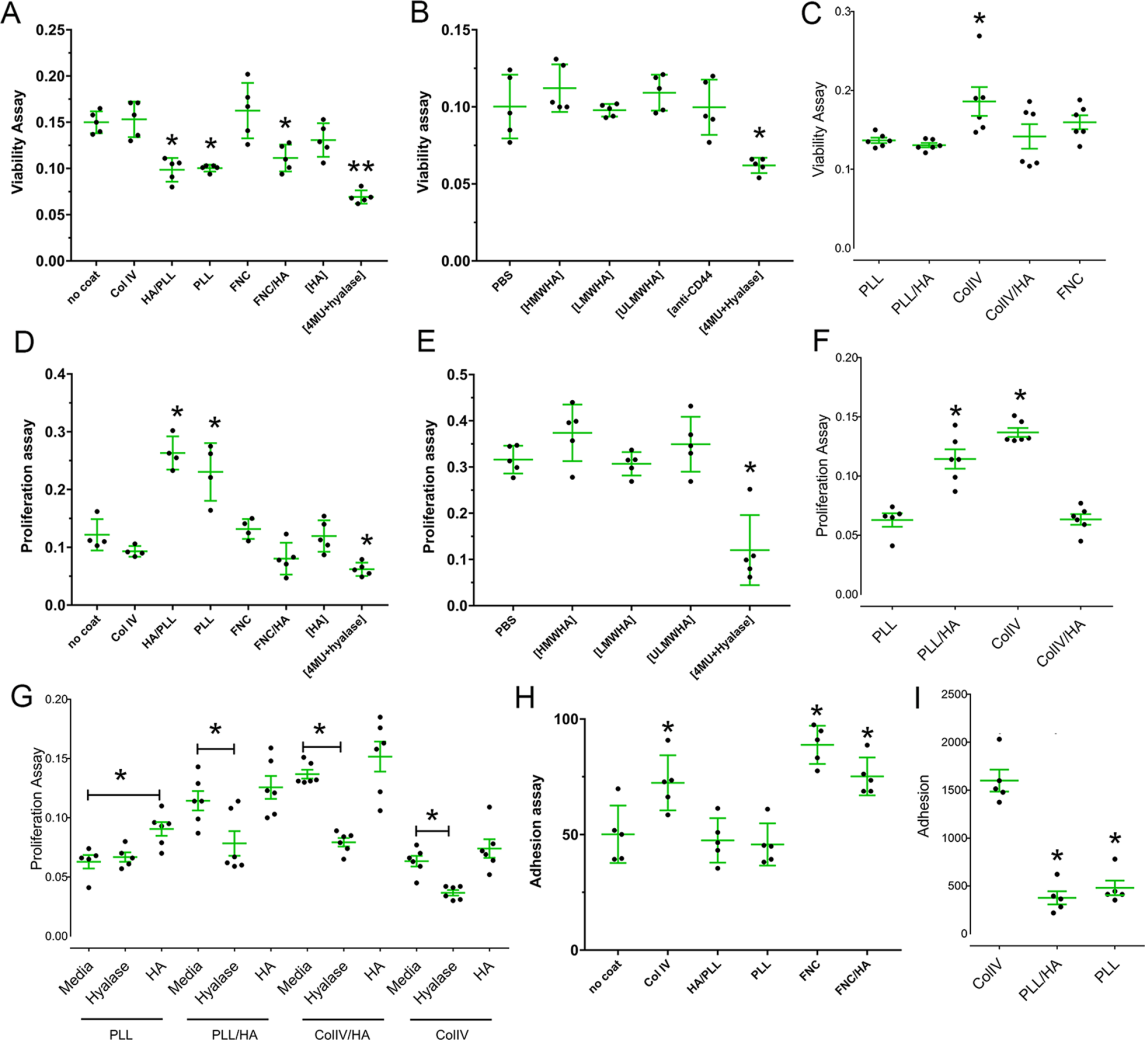
Effects of HA on viability, proliferation and adhesion of LESCs. TKE2 cells (**A**, **B**, **D**, **E** and **H**) and hLESCs (**C**, **F**, **G** and **I**) were maintained of differently coated culture dishes or in the presence of HA as a supplement to the media and the viability assayed using the MTT assay, proliferation assayed with a BrdU assay and adhesion assayed using an adhesion assay. *Represents *p* ≤ 0.05

Given LESCs express a rich HA matrix both in vivo and in vitro, and that it is vital for maintaining LESCs in vivo, we went on to investigate whether the endogenous HA matrix supports LESCs during ex vivo expansion. For such, hLESCs and TKE2 cells were treated or not with hyalase and 4MU in order to cleave endogenous HA and to prevent the synthesis of HA, respectively ([4MU + Hyalase]). The treatment of hLESCs and TKE2 cells with [4MU + Hyalase] significantly decreased their viability when compared to all other culture conditions (Fig. 2A, B). We also investigated whether exogenous HA could play a beneficial role in supporting hLESCs during ex vivo expansion. For such, the viability of TKE2 cells and hLESCs was assayed following culture on the differently coated dishes or after the addition of HA to the culture media using a MTT assay. Col IV and FNC had no significant effect on the viability of TKE2 cells when compared to the uncoated plates (Fig. 2A). However, PLL, PLL/HA and FNC/HA significantly reduced the viability of TKE2 cells from ~ 0.15 in the uncoated dishes to ~ 0.1 in PLL, PLL/HA and FNC/HA coated dishes (Fig. 2A). Exogenous HA supplemented in the media ([HA]) had no effect on the viability of TKE2 cells (Fig. 2A). In order to evaluate the effects of HA of different molecular weights on the viability of TKE2 cells, they were treated or not with HMWHA, LMWHA, ULMWHA, and, thereafter, cell viability was assayed. Curiously, HA of different molecular sizes had no effect on the viability of TKE2 when added to the culture media (Fig. 2B). TKE2 cells express high levels of CD44 (Additional file1: Fig. S1), the major cell surface receptor for HA. Therefore, anti-CD44 was also used to abrogate any effects of HA that could be mediated through binding to the CD44 receptor, however blocking this binding also had no effect on TKE2 cell viability (Fig. 2B). The suitability of using HA as a coat for maintaining hLESCs was also investigated by comparing the viability of hLESCs maintained on PLL/HA coated dishes when compared to other commonly used coats, for example ColIV and FNC. hLESCs cultured on ColIV coated dishes presented an increase in viability when compared to the other coated dishes (Fig. 2C).

### Effect of HA on proliferation of LESCs

TKE2 cells and hLESCs were seeded on the differently coated dishes or subjected to different treatments for 48 h, and, thereafter, subjected to BrdU incorporation for the final 8 h in order to evaluate their effect on cell proliferation. PLL and PLL/HA significantly promoted the proliferation of TKE2 cells by two fold, when compared to all other coated dishes (Fig. 2D). HA when added to TKE2 cells as a supplement to the media, had no effect on proliferation ([HA]—Fig. 2D). The treatment of TKE2 cells with 4MU and Hyalase ([4MU + Hyalase]), in order to cleave endogenous HA, significantly decreased the proliferation of TKE2 cells (Fig. 2D). The effects of HA of different molecular weights on TKE2 cell proliferation were also investigated by adding HMWHA, LMWHA or ULMWHA to the culture media of TKE2 cells, and, thereafter, cell proliferation was assayed (Fig. 2E). Curiously, soluble exogenous HA of different molecular sizes had no effect on the proliferation of TKE2 cells (Fig. 2E). The suitability of using HA as a coat for maintaining hLESCs was also investigated by comparing the proliferation of hLESCs maintained on PLL/HA coated dishes to ColIV and ColIV/HA. hLESCs maintained on PLL/HA and ColIV coated dishes presented an increase in cell proliferation when compared to PLL and ColIV/HA coated dishes (Fig. 2F). We also analyzed the effects of endogenous HA and soluble exogenous HA on hLESCs. In order to assess the effects of endogenous HA and exogenous soluble HA on hLESC proliferation, the cells were treated or not with Hyalase or HMWHA (Fig. 2G). Hyalase treatment significantly decreased the proliferation of hLESCs maintained on PLL/HA, ColIV/HA and ColIV coated dishes, thus cleaving the extracellular HA reduces the proliferation of hLESCs (Fig. 2G). Exogenous soluble HA solely promoted proliferation of hLESCs maintained on PLL coated dishes and had no effect on hLESCs maintained on the other coated dishes (Fig. 2G).

### Effect of HA on the adhesion of LESCs

In order to compare the adhesion properties of TKE2 cells on the different substrates, the cells were seeded on uncoated dishes (control) or culture dishes pre-coated with either ColIV, PLL, PLL/HA, FNC or FNC/HA, left to adhere for 60 min, non-adherent cells removed, and the number of adhered cells manually counted. TKE2 cells presented a significant increase in the number of adhered cells on FNC, FNC/HA and Col IV coated dishes, when compared to the uncoated dishes (Fig. 2H). In contrast, PLL and PLL/HA had no effect on the adhesion of TKE2 cells when compared to uncoated dishes (Fig. 2H). There was a slight decrease in the number of adhered cells to the FNC/HA coated dishes when compared to the FNC coated dishes, however this did not reach significance. We also analyzed the effects of differently coated dishes on the cell adhesion of hLESCs. There was a significant reduction in the adhesion of hLESCs onto PLL/HA and PLL coated dishes, when compared to ColIV coated dishes (Fig. 2I).

### Effect of HA on the migration of TKE2 cells

**Fig. 3:**
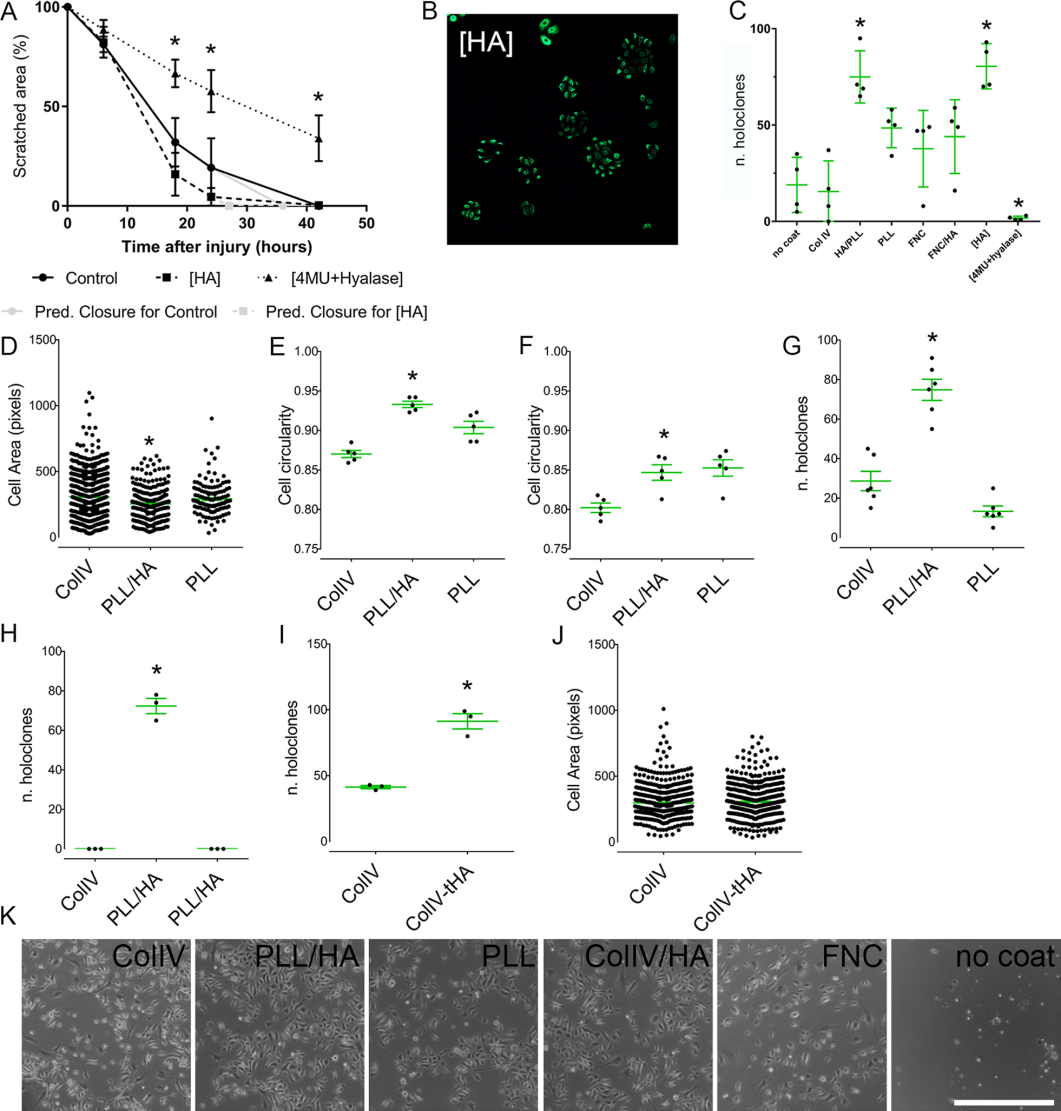
Effects of HA on the migration, cell size and colony formation capabilities of LESCs. TKE2 cells were maintained until confluence and, thereafter a scratch made through the center of the culture dish using a pipette tip, as previously shown [87] (**A**). A representative image of TKE2 cells accumulating in clusters instead of evenly distributing throughout the culture dish when maintained on PLL/HA coated dishes (**B**). The colony formation capabilities of TKE2 cells were assayed on differently coated dishes compared to uncoated controls and in the presence or not of Hyalase and 4MU (**C**). The number of holoclones that were larger than 1000 μm were counted (**C**). The cell area (**D**) and cell circularity (**E** and **F**) was quantified for hLESCs maintained on differently coated dishes. The colony formation capabilities of hLESCs were assayed when maintained on differently coated dishes in the presence (**G**) or absence of 3T3 cells (**H**). The colony formation capabilities (**I**) and cell area (**J**) of hLESCs were assayed after being maintained on ColIV or on ColIV followed by PLL/HA. Representative images captured using an EVOS microscope evidencing the morphology of hLESCs maintained of the differently coated dishes (**K**). *Represents *p* ≤ 0.05, scale bar represents 1000 μm

In order to study whether HA, either exogenous or endogenous, promotes TKE2 cell migration, an in vitro scratch assay was used. After the scratch was made either HA, 4MU + Hyalase or PBS (vehicle control) was added to the cells, and the cells allowed to migrate into the gap for 40 h. Exogenous HA significantly promoted the migration of TKE2 cells with complete wound closure predicted to be at ~ 27 h, compared to ~ 36 h in the control (Fig. 3A). 4MU + Hyalase significantly inhibited the migration of TKE2 cells, with ~ 40% of the scratch area remaining at 40 h (Fig. 3A).

### HA promotes the formation of colonies in TKE2 cells culture

A colony formation assay (Fig. 1C) was used as a means to evaluate the stem cell-like properties of TKE2 cells. For such, TKE2 cells were seeded at a low density on differently coated dishes or subjected to different treatments and the number of colonies counted after 12 days. Seeding the cells on PLL/HA coated dishes and the addition of exogenous HA both significantly promoted the formation of “holoclone-like” colonies by TKE2 cells when compared to all other coated dishes, uncoated dishes, and treatments (Fig. 3C). Interestingly, 4MU and Hyalase treatment, which in these experiments serves to cleave the endogenous HA matrix present surrounding the TKE2 cells, abrogated the colony formation capabilities of the TKE2 cells (Fig. 3C). Interestingly, the increased tendency for TKE2 cells to form colonies on PLL/HA was evident when seeding cells for all experiments. TKE2 cells seeded on HA coated dishes or when cultured in the presence of media supplemented with HA tended to accumulate into clusters of cells, thus, TKE2 cells readily formed colonies when cultured in the presence of HA (Fig. 3B). TKE2 cells cultured at 2.5 × 10^4^ cells/mL density in media supplemented with HA for 48 h, processed for immunocytochemistry and stained with ∆Np63 shows that in the presence of HA, TKE2 cells accumulate within an organized clusters of cells instead of homogenously spreading throughout the culture dish, as seen in the absence of HA (Fig. 3B).

Previous studies have demonstrated that within LESC preparations from the limbal rim, the fraction of cells with a smaller size have the highest clonogenic potential and have the highest expression levels of putative LESCs markers [51–53]. Thus, LESCs are believed to be smaller in size when compared to TACs, differentiated corneal epithelial cells and other cell types that are present in the limbal region. Thus, we used the cell area as a metric to evaluate hLESCs cultured on differently coated dishes. hLESC cultured on PLL/HA coated dishes presented a significantly smaller cell area when compared to cells cultured on ColIV and PLL (Fig. 3D). Studies have also demonstrated that within epithelial cell extracts obtain from corneal limbal rims, hLESCs have a rounder cell shape when compared to other cells [51, 54]. Thus, we determined the cell circularity of cells maintained of the differently coated dishes, with and without 3T3 cells at passage 3 (Figs. 3E, F respectively). For this assay, a perfectly round cell would have a circularity index of 1. hLESCs maintained on PLL/HA coated dishes presented significantly higher cell circularity when compared to cells seeded on ColIV and PLL in the presence of 3T3 cells (Fig. 3E), and ColIV in the absence of 3T3 cells (Fig. 3F). The ability of hLESCs to form holoclones on differently coated dishes was also evaluated both in the presence and absence of 3T3 feeder cells. There was a significant increase the in formation of “holoclone-like colonies” by hLESCs cultured on PLL/HA coated dishes when compared to ColIV and PLL coated dishes in the presence of 3T3 feeder cells (Fig. 3G). Interestingly, hLESCs cultured on PLL/HA cultured dishes in the absence of 3T3 cells were able to form “holoclone-like” colonies, while those cultured on ColIV and PLL were not (Fig. 3H). Given that our data demonstrated ColIV is a superior coat for promoting the adhesion of hLESCs following cell isolation, however, PLL/HA is the superior coat to maintain hLESCs in culture, we investigated using ColIV coated dishes for maintaining hLESCs following cell isolation, and, thereafter, maintaining the cells on PLL/HA coated dishes, namely ColIV-transferred onto PLL/HA (ColIV-tHA). hLESCs cultured on ColIV-tHA coated dishes presented a significant increase in the ability to form “holoclone-like” colonies when compared to those maintained on ColIV alone (Fig. 3I). The cell area of hLESCs cultured on ColIV was also compared to ColIV-tHA. There was no significant difference in the cell area of hLESCs when cultured on ColIV or ColIV-tHA coated dishes (Fig. 3J). Images of the cell morphology of hLESCs maintained on the differently coated dishes can be seen in Fig. 3K.

### Effect of different coats on the expression of putative LESC markers in TKE2 cells

**Fig. 4:**
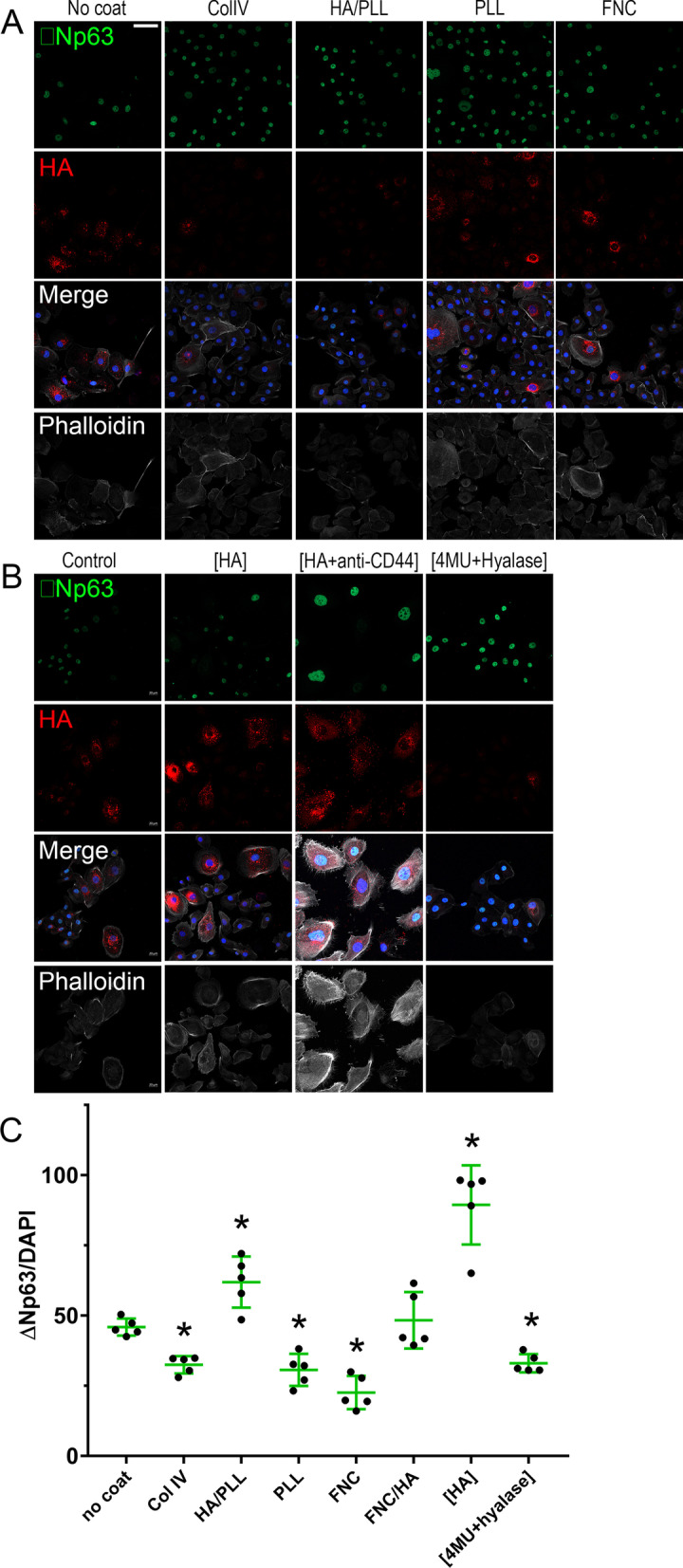
Effects of HA on TKE2 cells cultured on differently coated dishes or in the presence of HA as a supplement to the media. TKE2 cells were cultured on differently coated dishes (**A**) or on uncoated dishes with HA, HA + anti-CD44 or 4MU + Hyalase supplemented to the media (**B**). The number of ∆Np63 positive cells and the total number of cells (DAPI) were quantified using QuPath [88] and the percentage of ∆Np63^+^ cells calculated (**C**). *Represents *p* ≤ 0.05, scale bar represents 50 μm

The effect of the differently coated dishes on the expression profile of putative stem cell markers in TKE2 cells was also investigated. For such, TKE2 cells were seeded on differently coated dishes and the expression profile of the putative stem cell marker ∆Np63 analyzed via immunocytochemistry, representative images are presented in Fig. 4A. The total number of ∆Np63 and DAPI positive cells were quantified in five fields per experimental point using Qupath [55], the experiment was carried out in triplicate and repeated four times. The number of ∆Np63 and DAPI positive cells were presented as the number ∆Np63 positive cells per total number of cells (Fig. 4C). The number of cells expressing the LESC marker ∆Np63 was significantly increased in TKE2 cells cultured on HA coated dishes and cultured in the presence of HA supplemented in the media, specifically, PLL/HA and FNC/HA coated dishes, and, also, media supplemented with HA ([HA]) when compared to TKE2 cells maintained on uncoated dishes (Fig. 4A, B). Culturing TKE2 cells on Col IV, PLL and FNC significantly decreased the number of cells expressing the putative stem cell marker ∆Np63 when compared to uncoated dishes (Fig. 4A, C). TKE2 cells treated with [4MU + Hyalase] presented a significant reduction in the number of cells expressing the putative stem cell marker ∆Np63 (Fig. 4B, C).

### Effect of differently coated dishes and HA on the differentiation of TKE2 cells

**Fig. 5:**
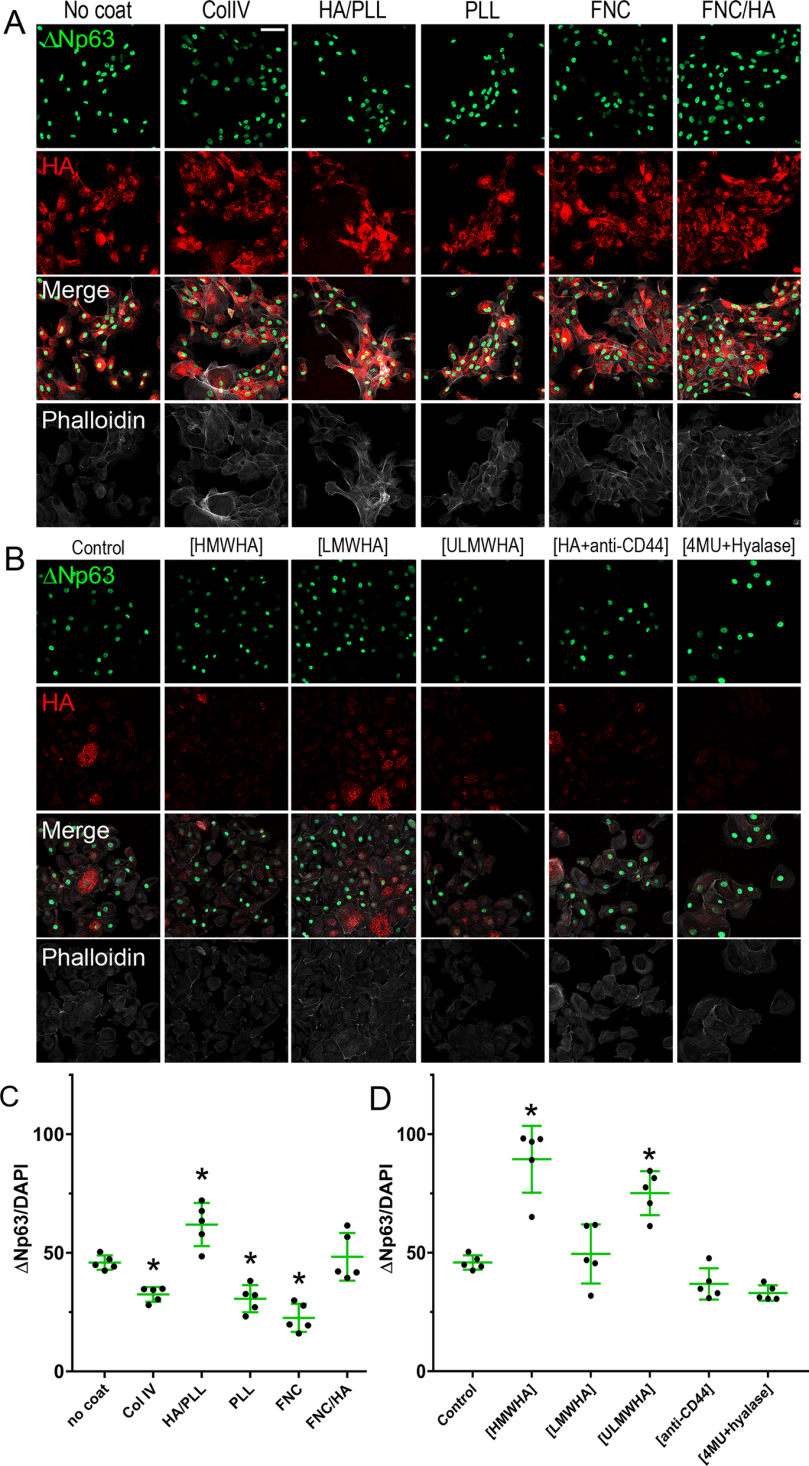
Effects of HA on the differentiation of TKE2 cells cultured on differently coated dishes or in the presence of HA as a supplement to the media. TKE2 cells were cultured on differently coated dishes (**A**) or on uncoated dishes with HA of different molecular weights, HA + anti-CD44 or 4MU + Hyalase supplemented to the media (**B**) under differentiating conditions. The number of ∆Np63 positive cells and the total number of cells (DAPI) were quantified using Qupath [88] and the percentage of ∆Np63^+^ cells calculated for TKE cells maintained on differently coated dishes (**C**) and with HA, HA + anti-CD44 and 4MU + Hyalase supplemented to the media (**D**). *Represents *p* ≤ 0.05, scale bar represents 50 μm

Given the increase in the number of ∆Np63 positive cells when TKE2 cells were cultured in the presence of HA, either as a coat or supplemented in the media, we investigated whether HA could prevent the differentiation of TKE2 cells. For such, TKE2 cells were seeded on differently coated dishes and/or in media supplemented with HA of different sizes, [anti-CD44] or [4MU + Hyalase] and subjected to conditions that have been shown to promote the differentiation of TKE2 cells [46, 52]. The number of ∆Np63 positive cells was estimated as outlined above. Interestingly, TKE2 cells cultured in the presence of HA, specifically, PLL/HA and FNC/HA coated dishes, and, also, media supplemented with HA, namely [HA], presented significantly more ∆Np63 positive cells, indicating HA prevented limbal epithelial progenitor cell differentiation in TKE2 cultures (Fig. 5A, C). Interestingly, culturing TKE2 cells on ColIV, PLL and FNC significantly reduced the expression of the putative stem cell marker ∆Np63, indicating these coats do not provide any protective role in regard to preventing LESCs differentiation and actually promote the differentiation of TKE2 cells when compared to uncoated dishes (Fig. 5A, C). TKE2 cells treated with [4MU + Hyalase] also presented a significant reduction in the expression of the putative stem cell marker ∆Np63, indicating the endogenous HA matrix is able to maintain the TKE2 cells in a more undifferentiated state (Fig. 3B, C). The effect of HA of different sizes in preventing LESC differentiation in TKE2 cultures was also investigated. Interestingly, solely [HMWHA] and [ULMWHA] were able to prevent the loss of ∆Np63 positive cells when TKE2 cells were cultured in differentiation media (Fig. 5B, D).

### Effect of different coats on the expression of putative LESC markers in hLESCs

**Fig. 6:**
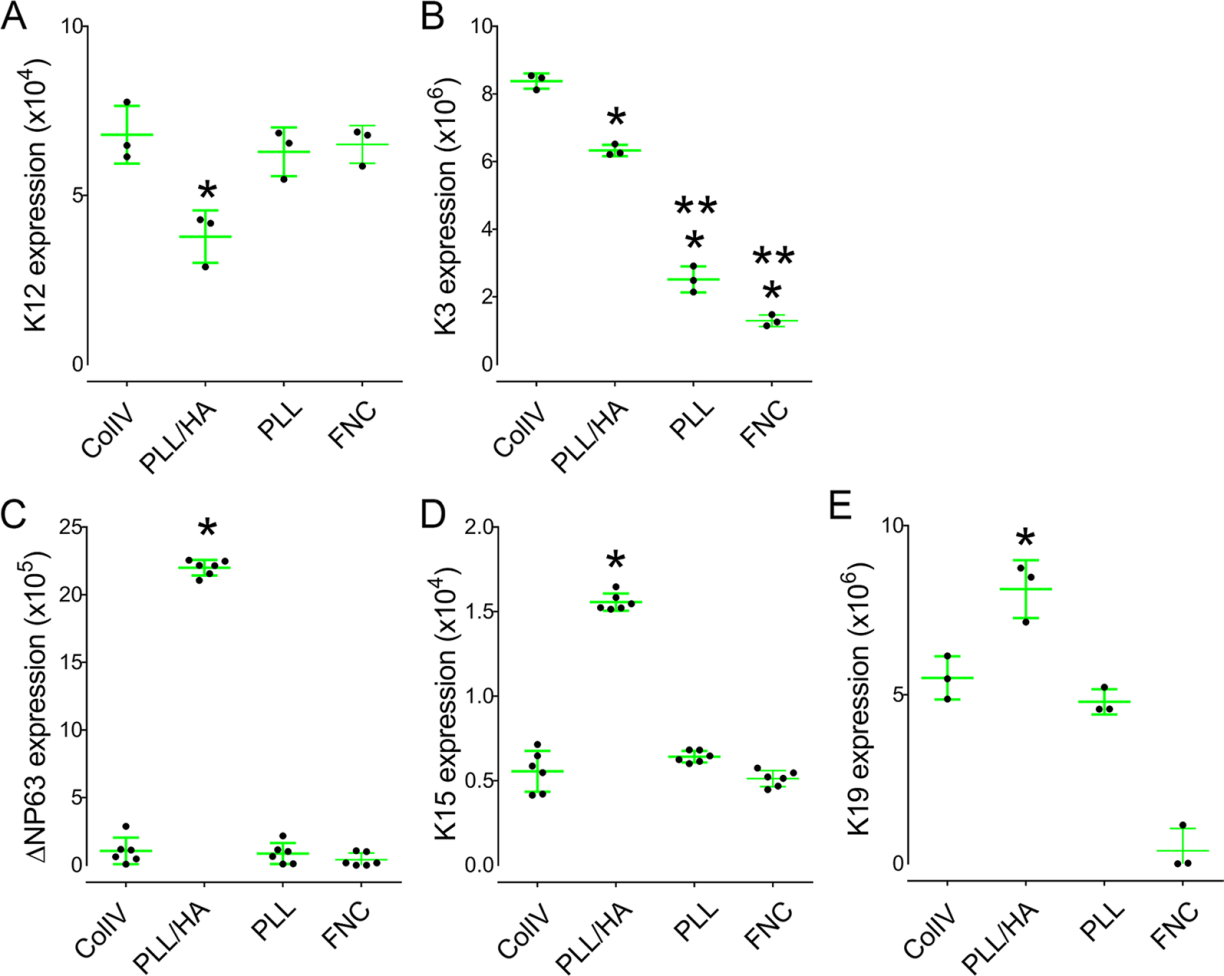
Effects of HA on the expression profile of putative stem cells markers by hLESCs. hLESCs were cultured on differently coated dishes and the expression profile of K12 (**A**), K3 (**B**) ∆Np63 (**C**), K15 (D) and K19 (**E**) investigated by real-time PCR (qPCR). *Represents *p* ≤ 0.05 in comparison with HA and **Represents *p* ≤ 0.05 for PLL and FNC in comparison with ColIV

**Fig. 7:**
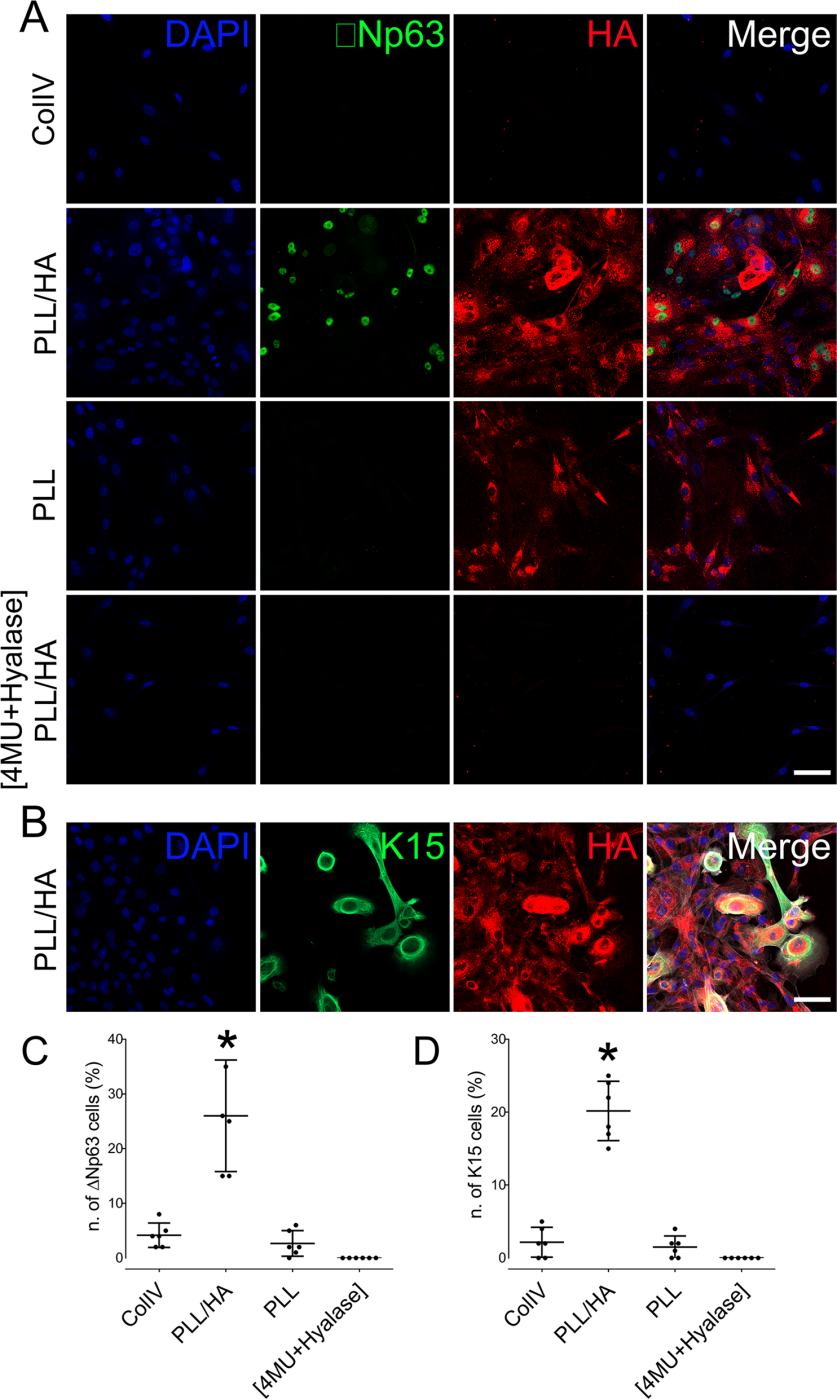
Effects of HA on the expression of ∆Np63 and K15 by hLESCs. hLESCs were cultured on differently coated dishes and the expression and localization of ∆Np63 (**A**) and K15 (**B**) investigated by immunocytochemistry. Scale bare represents 50 μm. The number of ∆Np63^+^ cells (**C**) and K15^+^ cells (**D**) was counted. *Represents *p* ≤ 0.05 in comparison with ColIV

hLESCs were seeded on either ColIV, PLL, PLL/HA or FNC and the expression levels of keratin 3 (K3), keratin 12 (K12), ∆Np63, keratin 15 (K15) and keratin 19 (K19) investigated by qPCR (Fig. 6A–E). hLESCs seeded on PLL/HA coated dishes presented a significant decrease in the expression levels of K12, when compared to ColIV, PLL and FNC (Fig. 6A), and a significant decrease in K3 expression when compared to ColIV (Fig. 6B). Interestingly, hLESCs seeded on PLL/HA coated dishes presented a significant increase in the expression levels of putative stem cell markers ∆Np63, K15 and K19, when compared to ColIV, PLL and FNC (Fig. 6C–E). The expression of putative stem cell markers in hLESCs was also verified by immunocytochemistry. Interestingly, a drastic increase in the number of ∆Np63 and K15 positive cells can be seen when hLESCs are cultured on PLL/HA cultured dishes when compared to Col IV and PLL coated dishes (Fig. 6A–D). Less than 5% and 2% of total cells within the hLESCs cultures that were seeded on either ColIV or PLL presented ∆Np63 and K15 staining, respectively, whereas ~ 25% and 22% of cells seeded on PLL/HA presented ∆Np63 and K15 staining, respectively (Fig. 7C, D). Interestingly, there was also a significant increase in the number of cells presenting an HA-rich glycocalyx when cells were seeded on PLL/HA coated dishes (Fig. 7A). As seen throughout our study, cells presenting an HA glycocalyx strongly correlated with the cells that expressed putative LESC markers. Treatment with 4MU + Hyalase prevented the assembly of an HA-rich glycocalyx, and consequently ∆Np63 and K15 positive cells were no longer present (Fig. 7C, D).

### Effect of different coats on senescence of hLESCs

**Fig. 8:**
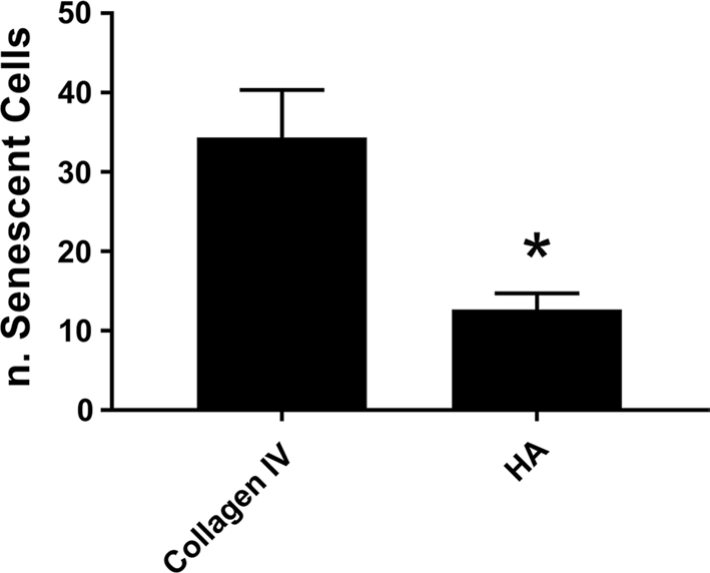
Effects of HA on hLESC senescence. hLESCs were cultured on ColIV or PLL/HA and cell senescence assayed using the Senescence *β*-Galactosidase Staining Kit (#9860S, Cell Signaling Technology). Cells were analyzed under a microscope and the number of senescent cells counted. *Represents *p* ≤ 0.05

hLESCs were maintained on either ColIV or PLL/HA and maintained for eleven passages. Cell senescence was assayed at each passage from passage 6, and at passage 11, senescent cells were evident in LESCs cultures maintained on ColIV and PLL/HA coated dishes; however, significantly more senescent cells were present in LESCs maintained on ColIV coated dishes when compared to cells maintained on PLL/HA coated dishes (Fig. 8). Thus, seeding hLESCs on HA can reduce the number of cells entering senescence over time.

## Discussion

The specific composition of the ECM in the LESC niche provides the necessary microenvironment for maintaining healthy and viable LESCs in vivo [38, 56]. Our group previously showed that the LSCN is composed of a specialized HA matrix, and that the loss of this matrix, in mice lacking HA synthase 2 in keratin 14 expressing cells, also lack LESCs [38]. HA is a non-sulfated glycosaminoglycan component of the extracellular matrix, consisting of repeating disaccharide subunits composed of N-acetylglucosamine and *β*-glucuronic acid. HA exists in tissues in primarily two forms: high-molecular weight HA (HMWHA) and low molecular weight HA (LMWHA). HMWHA has been shown to play an important role in development, in homeostasis and is an integral component of various stem cell niches [57–60], whereas LMWHA and smaller HA fragments (such as ultra-low molecular weight HA (ULMWHA)) are primarily correlated with pathogenesis and have been shown to have pro-inflammatory properties in tissues [61, 62]. Human umbilical cord mesenchymal stem cells (hUMSCs) have been shown to express an HA-rich glycocalyx, both in vivo and in vitro, and when this HA matrix is cleaved from the cells prior to transplantation, they are rejected [34]. Thus, we speculated LESCs also express an HA-rich matrix in vitro which could provide a niche-like environment for these cells while being cultured ex vivo. Therefore, we investigated whether LESCs express a HA-rich matrix in vitro and, thereafter, if this endogenous HA supports LESCs during ex vivo expansion. Interestingly, we found that hLESCs and TKE2 cells both produce a significant amount of HA in vitro which is assembled into a net-like matrix surrounding the LESCs, as seen in vivo [38]. We thereafter attempted to investigate whether this HA matrix produced by LESCs in vitro could play a role in supporting the stem cell phenotype during ex vivo expansion, as seen in vivo. For such, the endogenous HA matrix surrounding LESCs was removed by enzymatic digestion and a chemical inhibitor was used to prevent further HA synthesis. Interestingly, we found that removing this specific HA-rich matrix in vitro drastically reduced the viability, proliferation, migration, colony formation capability and overall number of LESCs (based on the expression of putative stem cell markers). Thus, LESCs produce an HA-rich microenvironment in vitro which supports them during ex vivo expansion. Thus, our data show that endogenous HA helps to maintain viable LESCs during ex vivo expansion. CD44 is a ubiquitous, transmembrane glycoprotein receptor which binds to ECM ligands, such as HA, collagen and fibronectin and mediates various cellular functions such as growth, motility and inflammation [63–66]. Given that CD44 is the major cell surface receptor for HA and that we have previously found that CD44 is expressed throughout the corneal and limbal epithelium [38], we investigated whether CD44 could mediate the effects of HA on LESCs. Our data indicate that CD44, at least in part, mediates the effects of endogenous HA in LESCs. CD44 has been previously shown to be a prominent marker for cancer stem cells and HA-CD44 interactions mediate self-renewal and maintenance of these cells [67, 68]. We speculate similar interactions could be involved in the maintenance of LESCs in in vivo and in vitro, however further research is required.

Given the important role the endogenous HA microenvironment plays both in vivo and in vitro, we speculated that providing exogenous HA in vitro could be beneficial during ex vivo expansion of LESCs. Therefore, we investigated the use of HA as a substrate and supplement to the media for supporting both mouse progenitor cells (TKE2 cells) and human LESCs (hLESCs) ex vivo. Overall, our data indicated that HA coated dishes promotes LESC proliferation, migration, colony formation potential of LESCs, while preventing their differentiation, both in hLESCs and TKE2 cells. However, LESCs maintained on HA coated dishes presented reduced viability and adhesion when compared to ColIV and uncoated dishes. LESCs are quiescent in vivo, however a characteristic of LESCs is their high proliferative capabilities during corneal wound healing and/or during ex vivo culture [6, 69]. Therefore, high proliferative potential is an important characteristic of LESCs enabling a high number of LESCs to be obtained from small limbal biopsies for LESC transplantation [70–72]. The colony formation assay has widely been used as a surrogate assay to evaluate the “stemness” of stem cells [37, 73]. In principal, stem cells produce holoclones that are capable of extensive proliferation and self-renewal [74, 75]. Thus, a higher number of holoclones is a strong indication of stem cell enrichment within the LESC cultures, which is important in LESC expansion for transplantation [76]. In order to further investigate the effects of HA on LESCs, we analyzed the expression levels of LESC markers after culturing hLESCs and TKE2 cells on differently coated dishes or in media supplemented with different forms of HA. Our data show that culturing LESCs on HA coated dishes promotes the expression of LESC putative stem cell markers, while decreasing the expression levels of differentiated cornea epithelial cell markers. Thus, our results show that HA coated dishes supports the LESC phenotype during ex vivo expansion. Thus, the fact that maintaining LESCs on HA coated dishes promotes proliferation, supports the colony formation potential, and prevents the differentiation of LESCs indicates HA is a good candidate as a substrate for maintaining LESCs ex vivo.

Over the years, various studies have been dedicated to identifying specific ECM components in vivo that could be used to support LESCs in vitro [18, 77–79]. Studies have successfully identified various components that are differentially expressed in the limbus compared to the central cornea, such as laminin α1, α2, β1 chains, agrin, tenascin-C and HA [38, 80]. Several of these compounds have also been evaluated in vitro either as a substrate or as a supplement in culture media to promote the survival and proliferative capacity of the LESCs [78, 79]. Although many of these studies indicated certain ECM components support LESCs ex vivo, these compounds were only able to partially support different properties and functions of LESCs. Polisetti et al. [78] showed that laminin-511/521 isoforms enhanced LESC adhesion, migration and proliferation but did not affect differentiation, whereas laminin-332 suppressed proliferation but promoted adhesion, migration and LESC marker expression. Agrin added as a supplement to the culture media promoted the proliferation of LESCs and increased LESC marker expression but other LESC characteristics were not evaluated in vitro [79]. These studies indicate that a combination of LESC niche constituents could be used to provide significant improvement over currently used substrates for LESC culture and expansion. Our data indicate that ColIV is a superior coat for promoting the adhesion of LESCs, while HA is a superior coat for maintaining LESCs. Thus, we investigated using ColIV as an initial coat for seeding single cell suspensions isolated from limbal epithelial sheets, and, thereafter, transferring the cells onto PLL/HA coated dishes for maintaining the cells (ColIV-tHA). With this, we were able to obtain a higher yield of cells with increased colony formation capabilities when compared to ColIV and PLL/HA alone.

Currently, culturing cells on amniotic membranes have shown the greatest success in maintaining healthy viable LESCs during ex vivo cultures for CLET in the clinic [29, 30]. The Tseng group has shown that the amniotic membrane is an HA-rich tissue and the HA complexes within the amniotic membrane support the survival of LESCs both in vivo, as well as, ex vivo [31]. HA hydrogel scaffolds, which provide a 3D microenvironment for the cells and eliminates the attachment issues of HA have been reported to support different types of stem cells [55, 81, 82]. HA hydrogel scaffold-based culture system has also been recently used for xeno-free culture of human corneal epithelial stem cells [83]. In this current study, we analyzed the role of HA, both exogenous and endogenous, in maintaining mouse corneal epithelial progenitor cells and primary human LESCs ex vivo. For such, we analyze the efficacy of using HA as a substrate in comparison to other commonly used substrates for culturing LESCs. When testing the physiological properties of substrates for culturing cells, studies generally use solely uncoated plastic culture plates as a control, however, in our study we included commonly used substrates, such as collagen IV and FNC, as additional controls since we found LESCs do not adhere well to uncoated culture dishes. It is also important to note that when isolating LESCs from the limbal region, the cells obtained are not exclusively made up of LESCs, but instead also include melanocytes, transient amplifying cells (TACs), some differentiated epithelial cells and potentially a few resident inflammatory cells [84]. Moreover, LESCs primarily undergo asymmetric division, thereby, producing one LESCs and one TACs, however they also may undergo symmetric division, producing either two LESCs or two TACs [8, 85]. In our study HA could either be promoting the survival of exclusively LESCs. Alternatively, HA could be promoting the symmetric division of LESCs, favoring the production of two LESCs, and thereby enhancing the preponderance of LESCs in the cultures. Finally, HA could be simply inhibiting the differentiation of LESCs/TACs into corneal epithelial cells. Currently no good markers are available for identifying TACs, and it is believed that TACs express many of the putative LESC markers [86]. Thus, in our study we have not been able to distinguish between LESCs and TACs within our LESC cultures using exclusively LESC markers. However, studies have shown that holoclones are formed exclusively by LESCs, and that TACs instead form meroclones [51, 52].Thus, based on the colony formation data, our findings suggest that HA supports LESCs.

Taken together, this study shows that HA is an important component of the LESC niche both in vivo and in vitro. Moreover, this HA niche aids in maintaining viable LESCs in vitro, supporting our previously published data in vivo [38]. Our data also show that exogenous HA can be used to support LESCs ex vivo. Importantly, our data indicate that HA is capable of supporting LESCs in the presence and absence of 3T3 feeder cells, which makes HA a more translationally relevant coat. HA is relatively cheap, has very flexible storing conditions and is quick and easy to prepare, and thus, would be very easy to be implemented for use in coating culture dishes and/or to be added to the media of LESCs to help support LESCs during ex vivo expansion. Further studies are necessary to understand the mechanism by which both endogenous and exogenous HA maintain LESCs. Future studies could be directed toward characterizing the HA matrix of LESCs in the clinic during the ex vivo expansion, and, thereafter, this data could be correlated with transplantation success. In time, this data could be used to generate quality control guidelines for LESCs using the characteristics of the HA matrix surrounding LESCs during ex vivo expansion as a predictor for transplantation success.

## Conclusions

HA is a vital component of the LESC niche, both in vivo and in vitro, and is necessary for maintaining viable LESCs [38]. In addition to the endogenous HA that is produced by LESCs both in vitro and in vivo, exogenous HA can be added to further support LESCs during ex vivo expansion. Together the increased colony forming ability and increase in the expression of stem cell markers in the presence of PLL/HA indicates HA is a superior coat at maintaining LESCs during ex vivo expansion than the commonly used ColIV. Importantly, our data indicate that HA is capable of supporting LESCs in the presence and absence of 3T3 feeder cells, making HA a translationally relevant coat. Overall, HA is cheap, has very flexible storing conditions and is quick and easy to prepare, and thus, would be very easy to be implemented for use in coating culture dishes and/or to be added to the media of LESCs to help support LESCs during ex vivo expansion around the world. The use of HA to generate optimal LESC culture conditions represents a promising strategy to improve the success rate of LESC transplantation.

## Supplementary Information

**Additional file 1.** **Figure S1**: Expression of CD44 by TKE2 cells cultured on differently coated dishes. TKE2 cells were cultured on differently coated dishes and CD44 expression (green) analyzed by immunocytochemistry. Cells were counter stained with Phalloidin (white) and DAPI (blue) to evidence the cytoskeleton and nuclei, respectively. Scale bar represents 20 µm.

## Data Availability

Not applicable.

## Abbreviations

HA: Hyaluronan
LSCN: Limbal stem cell niche
LESCs: Limbal epithelial stem cells
hLESCs: Human limbal epithelial stem cells
TAC: Transient amplifying cell
LSCD: Limbal stem cell deficiency
CLET: Cultivated limbal epithelial transplantation
HLA: Human leukocyte antigen
hUMSCs: Human umbilical cord mesenchymal stem cells
LSCN: Limbal epithelial stem cell niche
ECM: Extracellular matrix
HBSS: Hanks’ balanced salt solution
DMEM: Dulbeco’s modified eagle medium
SHEM: Dulbeco’s modified eagle medium/Nutrient mixture F-12
FBS: Fetal bovine serum
ITS: Insulin transferrin selenium
EGF: Epidermal growth factor
dKSFM: Defined keratinocyte serum-free media
HABP: Biotinylated HA binding protein
Hyalase: Hyaluronidase from *Streptomyces hyalurolyticus*
Hyalase + 4MU: 1MM 4MU and 3U/ml hyalase
ColIV: Collagen IV
FNC: Fibronectin
PLL: Poly-l-lysine
PLL/HA: Poly-l-lysine with 0.1% hyaluronan
FNC/HA: Fibronectin with 0.1% hyaluronan
ColIV/HA: Collagen IV with 0.1% hyaluronan
HMWHA: High molecular weight hyaluronan
LMWHA: Low molecular weight hyaluronan
ULMWHA: Ultra-low molecular weight hyaluronan
MTT: 3 Methylthiazolyldiphenyl-tetrazolium bromide
DMSO: Dimethyl sulfoxide
OD: Optical density
HCGS: Human corneal growth serum
SD: Standard deviation
ANOVA: Analysis of variance
ColIV-tHA: ColIV-transferred onto PLL/HA
K3: Keratin 3
K12: Keratin 12
K15: Keratin 15
K19: Keratin 19
