# Supplementary figures and images for: Hyaluronan supports the limbal stem cell phenotype during ex vivo culture
Source: Stem Cell Res Ther. 2022 Jul 30;13:384. doi: 10.1186/s13287-022-03084-8 (PMC9338506; doi:10.1186/s13287-022-03084-8)

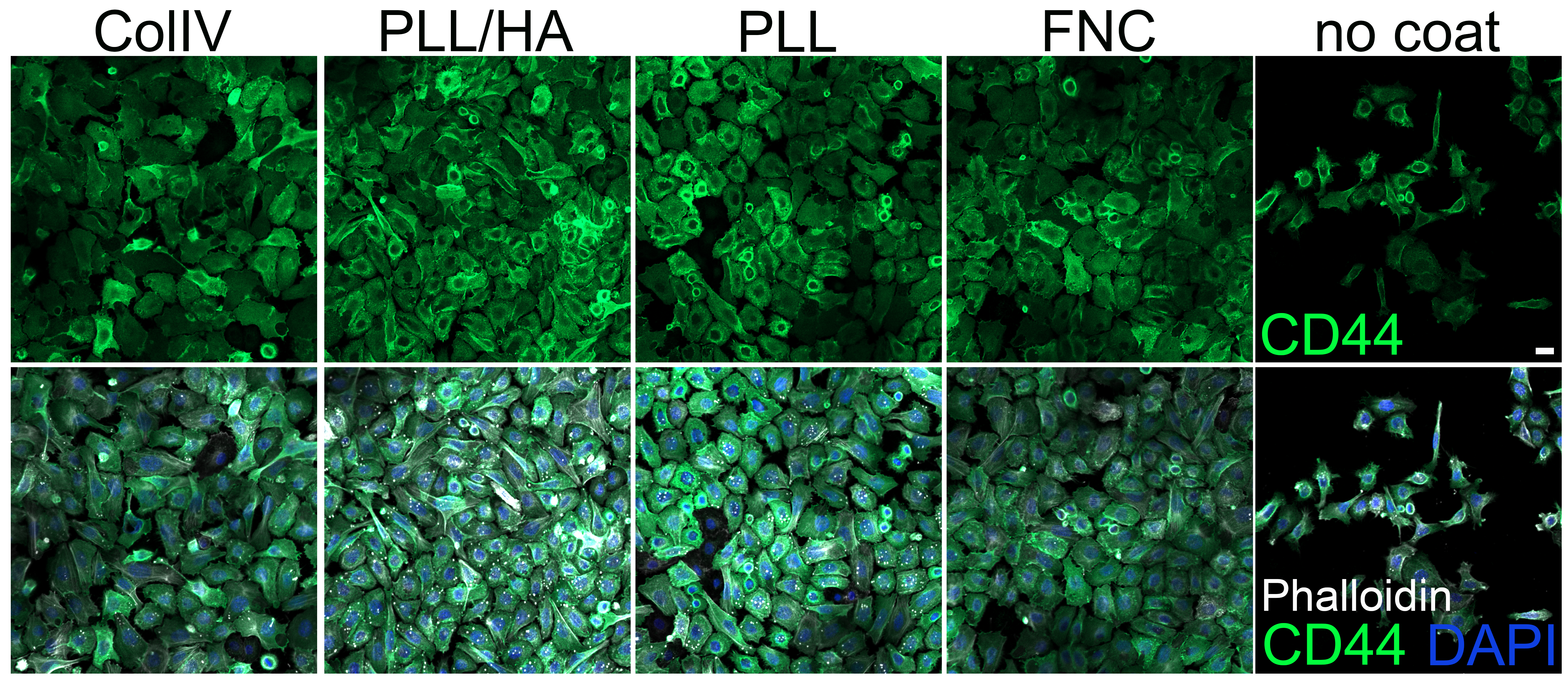

Supplement: Supplementary file 1 — Additional file 1. Figure S1: Expression of CD44 by TKE2 cells cultured on differently coated dishes. TKE2 cells were cultured on differently coated dishes and CD44 expression (green) analyzed by immunocytochemistry. Cells were counter stained with Phalloidin (white) and DAPI (blue) to evidence the cytoskeleton and nuclei, respectively. Scale bar represents 20 µm. [file 13287_2022_3084_MOESM1_ESM.tif]
